# Transcriptomic Convergence Reveals Tumour-Specific Stromal Remodelling in Immune-Enriched Papillary Thyroid Carcinoma

**DOI:** 10.3390/cancers18203406

**Published:** 2026-10-21

**Authors:** Veronika Petkova, Rumyana Dodova, Kalin Vidinov, Radka Kaneva

**Affiliations:** 1 Molecular Medicine Center, Department of Medical Chemistry and Biochemistry, Medical Faculty, Medical University of Sofia, 1431 Sofia, Bulgariakaneva@mmcbg.org (R.K.); 2 Department of Endocrinology, Medical Faculty, Medical University of Sofia, 1431 Sofia, Bulgaria

**Keywords:** papillary thyroid carcinoma, tumour microenvironment, cancer-associated fibroblasts, extracellular matrix remodelling, transcriptomics, nodular goitre

## Abstract

Background: Although immune activation can occur in both papillary thyroid carcinoma (TC) and nodular goitre (NG), the transcriptional programmes distinguishing malignant from non-malignant immune-rich thyroid tissue remain poorly understood. Methods: In this pilot study, bulk RNA sequencing of 19 specimens (10 TC, 9 NG) allowed classification into four tumour microenvironment (TME) subgroups. Results: Immune deconvolution showed that TC Immune-Enriched tumours were characterised by cancer-associated fibroblast (CAF) enrichment rather than predominant lymphocytic infiltration. Differential expression analysis identified only nine genes distinguishing TC Immune-Enriched from NG Immune-High tissue, suggesting transcriptomic similarity between malignant and benign immune-activated states in this small cohort. To identify tumour-associated stromal programmes, we identified genes shared between immune-enriched versus immune-depleted comparisons in papillary TC and nodular goitre. This yielded a 441-gene candidate tumour-associated stromal programme enriched for collagen-containing extracellular matrix organisation and TGFβ-associated ECM-remodelling pathways, supported by WGCNA and protein-protein interaction analyses. In 456 TCGA-THCA carcinomas, stromal enrichment correlated inversely with thyroid differentiation and was highest in BRAF-like tumours and the tall-cell histological variant. Conclusions: These findings suggest that transcriptomic convergence largely reflects shared inflammatory programmes, whereas stromal remodelling identifies a candidate tumour-associated stromal programme.

## Simple Summary

The thyroid gland can develop cancerous tumours and non-cancerous nodules, and both can show strong immune activity in the surrounding tissue. This similarity makes it difficult to tell at the molecular level whether immune-rich thyroid tissue is cancerous. We analysed gene activity in thyroid tissue samples and found that cancerous and non-cancerous immune-active tissues were very similar, differing in only nine genes, although the small sample size means we cannot rule out smaller differences. By comparing patterns across both tissue types, we instead identified a larger set of genes linked to changes in the tissue surrounding cancer cells. Tested against data from hundreds of independent thyroid tumour samples, this gene set was linked to more aggressive tumour features. These exploratory findings suggest that the tumour microenvironment, and particularly stromal remodelling, may provide information that is not captured by measures of immune activity alone and warrants further investigation as a potential biomarker of tumour biology.

## 1. Introduction

Thyroid nodules are among the most common endocrine conditions, with ultrasound detection rates of up to 68% in the general population. The prevalence is higher in iodine-deficient areas, and nodules are two to four times more common in females [1,2]. Despite this high prevalence, thyroid carcinoma accounts for only approximately 1% of human malignancies, and papillary thyroid carcinoma—the most common subtype—is present in roughly 5% of all thyroid nodules [3]. Although papillary TC typically follows an indolent clinical course, a subset of patients develops early lymphatic or distant metastases that significantly worsen prognosis [4]. In surgical cohorts, the prevalence of incidental thyroid carcinoma among patients undergoing thyroidectomy for nodular goitre can reach 12% in endemic regions—more than double the overall prevalence rate of 5.1% [5]. The challenge is reliably identifying this malignant minority among mainly benign cases.

Fine-needle aspiration biopsy remains the primary method for evaluating nodules before surgery. However, its sensitivity varies from 65% to 98%, and its specificity ranges from 72% to 100%, resulting in a significant number of nodules with indeterminate cytology [6]. This diagnostic grey zone—encompassing Bethesda categories III (AUS/FLUS), IV (FN/SFN), and V (SFM)—has driven the adoption of molecular testing in clinical practice. Both the American Thyroid Association and the revised Bethesda system recommend genetic analysis for indeterminate nodules, with particular focus on *BRAF* V600E and *RAS* driver mutations, as well as *RET/PTC* rearrangements and *PAX8::PPARG* fusions [7,8]. While mutation detection improves diagnostic accuracy, single-gene approaches do not fully capture tumour heterogeneity, and oncogenotype alone cannot predict the clinical behaviour of papillary TC.

Tumour behaviour is shaped not only by driver mutations but also by the tumour microenvironment (TME)—a dynamic ecosystem in which innate and adaptive immune cells, cancer-associated fibroblasts (CAFs), and endothelial cells interact with tumour epithelium via cytokines, chemokines, and extracellular matrix (ECM) components [9,10]. In thyroid cancer, the TME has received increasing attention, but its characterisation has remained largely descriptive. ESTIMATE-based analyses have demonstrated that TC samples exhibit variable stromal and immune scores, yet the transcriptional programmes underlying this heterogeneity—and whether they differ meaningfully between malignant and adjacent benign thyroid tissue—are poorly defined [11,12]. In particular, it is unknown whether immune infiltration in papillary TC reflects the same transcriptional state as immune activation in benign nodular disease, or whether cancer-associated stromal programmes exist that immune scoring alone does not capture.

The BRAF V600E mutation occurs in roughly 29–83% of papillary TC cases, depending on population and histological subtype [13]. In papillary TC, it is associated with more aggressive clinical features, such as extrathyroidal extension, lymph node metastasis, and lower radioiodine uptake. The Thyroid Differentiation Score (TDS), which measures the expression of thyroid-specific genes such as *NIS*, *TPO*, *TG*, and *TSHR*, is inversely related to tumour aggressiveness [14,15]. Although BRAF V600E is known to suppress differentiation markers through an autocrine TGFβ/Smad signalling loop, whether dedifferentiation in BRAF-driven papillary TC operates primarily through tumour-cell-intrinsic transcriptional reprogramming or through microenvironmental remodelling—particularly CAF activation and ECM deposition—remains unresolved. CAFs are now recognised as central mediators of tumour progression across multiple cancer types, remodelling the extracellular matrix, secreting pro-invasive growth factors, and creating an immunosuppressive niche that facilitates invasion and metastasis [16,17]. In thyroid cancer, stromal infiltration has been observed but not systematically characterised at the transcriptomic level, and its relationship to differentiation status and oncogenic identity remains formally unestablished [18].

Transcriptomic studies of the thyroid tumour microenvironment have compared carcinoma with non-malignant thyroid tissue or examined variation within carcinoma cohorts, including analyses using ESTIMATE-derived immune and stromal scores [19,20]. Nodular goitre has previously been included as a benign comparator in single-cell studies of PTC. Peng et al., for example, analysed tissues from eleven patients with PTC and three patients with nodular goitre, focusing on sex-associated differences in cellular composition and immune infiltration [21]. Our study addresses a different question: whether immune-enriched PTC is transcriptionally distinct from nodular goitre with comparable immune enrichment. We therefore aimed to distinguish carcinoma-associated transcriptional programmes from responses shared with immune-enriched benign tissue, including stromal programmes not resolved by aggregate immune scores.

Building on our previous targeted sequencing of thyroid nodules, which identified unexpected genomic overlap between nodular goitre and papillary TC [22], we performed a pilot, hypothesis-generating bulk RNA sequencing study of 19 surgically resected specimens, comprising both papillary TC and nodular goitre. Utilising ESTIMATE-based TME classification, immune deconvolution, differential expression analysis, and weighted gene co-expression network analysis, we asked (i) whether immune-enriched papillary carcinoma can be distinguished transcriptionally from immune-activated nodular goitre, and (ii) whether a candidate tumour-associated stromal programme can be identified beyond the shared inflammatory signal.

The resulting 441-gene programme was assessed for functional coherence through enrichment, co-expression and protein-interaction analyses, as well as for associations with differentiation, molecular and histological subtype, and clinicopathological features in 456 papillary TC samples from TCGA-THCA. Because TCGA-THCA is a tumour-only cohort, it cannot test the malignant–benign contrast.

## 2. Materials and Methods

### 2.1. Tissue Specimens

The research involved 9 patients with nodular euthyroid goitre and 10 patients with papillary thyroid carcinoma who received treatment at the Endocrine Surgery Clinic of the USBALE “Acad. Iv. Penchev” between 2017 and 2021, as shown in Table 1 and Table 2. Each participant provided written consent before their inclusion in the study. The study protocol was reviewed and approved by the ethics committee of the USBALE Acad. Iv. Penchev. Patients were assessed based on various clinical parameters: (1) sex; (2) type of hereditary thyroid pathology (malignant or benign); (3) histology of the cancer; (4) papillary TC subtype; (5) time of diagnosis (patient’s age); (6) tumour spread within the gland (multifocality) in malignant cases; (7) tumour size; (8) tumour spread to lymph nodes (lymphatic metastases); (9) presence of distant metastases; and (10) disease stage.

### 2.2. RNA Isolation

During surgery, half of the tissue was used for histopathological analysis, and the other half was stored at −80 °C. The total RNA from these tissues was extracted using the RNeasy Mini Kit (Qiagen, Hilden, Germany) following the manufacturer’s protocol, including DNase I treatment. The RNA integrity number (RIN) was assessed with an Agilent 2200 TapeStation System (Agilent Technologies, Santa Clara, CA, USA). Only samples with a RIN above 7.0 and free from signs of degradation were selected for subsequent RNA sequencing.

### 2.3. Bulk RNA Sequencing

Messenger RNA was enriched from 300 ng of total RNA using poly(T) oligo-attached magnetic beads (NEBNext^®^ Poly(A) mRNA Magnetic Isolation Module, New England Biolabs, Ipswich, MA, USA). Subsequently, unstranded RNA sequencing libraries were prepared using the NEBNext^®^ Ultra™ II RNA Library Prep Kit for Illumina^®^ (New England Biolabs, Ipswich, MA, USA) according to the manufacturer’s instructions. The libraries were sequenced on a NovaSeq 6000 (Illumina, Inc., San Diego, CA, USA) (2 × 150 bp). Raw sequencing reads were quality-filtered using FastQC v0.11, and low-quality reads and adapters were trimmed with Trimmomatic v0.39 [23,24]. Post-filtering quality assessment confirmed that at least 85% of bases had Phred quality scores of 30 or higher (Q30).

### 2.4. Read Alignment and Quantification

High-quality reads were aligned to the human reference genome GRCh38/hg38 using STAR v2.7.11 with default parameters for paired-end reads [25]. Multi-sample quality control with Qualimap v2.3 showed an average uniquely mapped read rate of 84.6% and approximately 20 million reads per sample [26]. Gene-level quantification was performed with featureCounts v2.0.3, achieving a 75% assignment rate for read pairs [27].

### 2.5. Immune Deconvolution

To characterise the immune microenvironment, we applied four complementary deconvolution algorithms to normalised TPM expression data. Overall immune and stromal infiltration were estimated using the ESTIMATE algorithm, which derives immune and stromal scores, as well as tumour purity, for each sample from curated gene signatures [20]. Samples were classified as immune-infiltrated or immune-depleted based on score distributions. Relative immune cell fractions were quantified using quanTIseq, which provides cell-type fractions for 11 immune populations [28]. EPIC was used to quantify major immune populations and CAFs [29]. To independently validate CAF abundance estimates, MCP-counter was used to estimate absolute abundance scores for eight immune and two stromal cell populations [30]. This combination of reference-based (quanTIseq, EPIC) and marker-based (MCP-counter) approaches enabled cross-validation and minimised bias.

Differential immune cell infiltration across TME subgroups was assessed using the Kruskal–Wallis test, with pairwise post hoc comparisons performed using the Dunn test. All *p*-values were corrected with the Benjamini–Hochberg (BH) false discovery rate method. Spearman’s rank correlation was used to analyse the relationship between ESTIMATE scores and cell fractions estimated through deconvolution. All statistical analyses were carried out in R v4.2.2 with the rstatix package. Visualisations were generated using ggplot2, ggpubr, and patchwork. *p*-values < 0.05 were considered statistically significant.

### 2.6. Differentially Expressed Genes (DEGs)

Differential expression analysis was conducted using DESeq2 v1.38.3 with default settings [31]. Lowly expressed genes were filtered out. Criteria for identifying differential expression included an |log2FC| ≥ 1 and a corrected *p*-value of ≤0.05. The BH method was employed to adjust *p*-values for multiple testing. Six pairwise contrasts were performed across the four TME subgroups (TC Immune-Enriched, TC-IE; TC Immune-Depleted, TC-ID; NG Immune-High, NG-IH; NG Immune-Low, NG-IL) using the Wald test. Visualisation of the differentially expressed genes was performed using the VolcanoPlot v3.5.1) and pheatmap v1.7.3 packages.

### 2.7. Intersection Analysis

To differentiate tumour-associated stromal programme from inflammation-driven transcriptional programme, we intersected upregulated DEGs from TC Immune-Enriched versus TC Immune-Depleted with those from NG Immune-High versus TC Immune-Depleted, using the VennDiagram R package v1.8.2. Genes found in both sets were designated as part of a shared inflammatory programme, while genes unique to TC Immune-Enriched were identified as a tumour-associated stromal programme. This set-difference approach depends on predefined thresholds and does not by itself establish differential expression between TC-IE and NG-IH.

### 2.8. Functional Enrichment

Over-representation analysis was performed using EnrichR v3.4 against the GO Cellular Component 2025 and Reactome 2022 databases [32,33,34]. Results were ranked by adjusted *p*-value, and the top 10 terms per database are reported [34]. Among the 441 genes, those annotated to the Collagen-Containing Extracellular Matrix term (GO:0062023) with log2FC ≥ 2 in TC-IE versus NG-IL (*p*adj < 0.05) were extracted (*n* = 22).

### 2.9. Weighted Gene Co-Expression Network Analysis (WGCNA)

Weighted gene co-expression network analysis was performed using the WGCNA R package v1.74 [35]. Before network construction, hierarchical clustering based on Euclidean distances identified 1 sample (NG74) as an outlier and excluded it, yielding a final cohort of 18 samples. Gene expression data were normalised using a variance-stabilising transformation (VST), as implemented in DESeq2, with the same gene-level filter applied as in the differential expression analysis. A signed co-expression network was constructed using the blockwiseModules function with a soft-thresholding power of β = 18, the lowest power that yielded a scale-free topology with R^2^ ≥ 0.80 (R^2^ = 0.829; mean connectivity = 56.2). Module eigengenes (the first principal component of each module) were correlated with four binary trait vectors representing TME subgroup membership (TC Immune-Enriched, TC Immune-Depleted, NG Immune-High, NG Immune-Low) and one continuous malignancy trait (TC = 1, NG = 0) using Pearson correlation. Statistical significance was assessed using an approximation to Student’s t-distribution. Hub genes were defined as those with the highest intramodular connectivity, quantified by module membership (MM; the Pearson correlation between gene expression and the module eigengene). For modules of biological interest, hub genes were identified as the top 20 genes ranked by MM. Gene significance (GS) was calculated as the Pearson correlation between individual gene expression and the trait vector of interest. The concordance between MM and GS was assessed using a scatter plot, confirming the biological relevance of the identified hub genes.

### 2.10. Thyroid Differentiation Score (TDS) and Single-Sample Gene Set Enrichment Analysis (ssGSEA)

Following the TCGA-THCA methodology, a TDS was calculated using the expression levels of 16 thyroid-specific genes: *DIO1*, *DIO2*, *DUOX1*, *DUOX2*, *FOXE1*, *GLIS3*, *NKX2-1*, *PAX8*, *SLC26A4*, *SLC5A5*, *SLC5A8*, *TG*, *THRA*, *THRB*, *TPO*, and *TSHR* [15]. For each gene, we centred expression values on the cohort median and computed TDS as the mean of these median-centred deviations across all 16 genes per sample. Higher TDS values indicate greater differentiation, whereas negative values indicate dedifferentiation, a state associated with poorer clinical outcomes and shorter progression-free intervals (PFIs).

Additionally, we performed ssGSEA using a 441-gene stromal signature. Enrichment scores were calculated using a random-walk algorithm; raw scores were retained to maintain biological interpretability and ensure cross-dataset comparability.

### 2.11. Protein-Protein Interaction Analysis

Protein-protein interactions were analysed with STRING v12.5 (https://string-db.org/), using Homo sapiens as the reference [36]. The tumour-associated stromal programme was submitted with official HGNC symbols, and genes without mapped STRING proteins were excluded. The full STRING network included curated database, experimental, co-expression, and text-mining evidence. Edges represented interaction confidence, with a high-confidence threshold of ≥0.700. No first- or second-shell interactors were added. Clustering was performed using the Markov Cluster Algorithm (inflation = 3), and enrichment was tested against a genome-wide background using STRING’s built-in analysis.

### 2.12. TCGA-THCA Evaluation Cohort

To assess the stromal programme in an independent carcinoma cohort, we retrieved RNA-seq counts and clinical metadata for 456 primary papillary thyroid carcinomas from TCGA-THCA using TCGAbiolinks [37]. The dataset comprised 456 papillary thyroid carcinoma samples with matched clinical and molecular annotations from the TCGA-THCA Pan-Cancer Atlas. Raw count data were normalised using a variance-stabilising transformation (VST) followed by ComBat batch correction to account for sequencing batch effects, yielding a final matrix of 24,645 genes across 456 samples.

### 2.13. Clinical and Molecular Associations

The stromal ssGSEA score was evaluated across clinically and molecularly relevant subgroups. Associations with molecular subtype were assessed using annotations accompanying the original TCGA-THCA study. Tumours were grouped as *BRAF* V600E-like, *RAS*-like, or “other,” following the supplied annotations [15]. Clinical associations were examined across histological subtypes (Classical, Follicular, Tall Cell), AJCC pathologic stage (I–IV), and extrathyroidal extension status. Additionally, the inverse relationship between stromal infiltration and thyroid differentiation was quantified by correlating the stromal ssGSEA score with the calculated TDS and by stratifying samples into TDS tertiles (low, mid, high differentiation) [15]. Receiver operating characteristic (ROC) curves were used to assess the ability of the stromal ssGSEA score and TDS to discriminate between established molecular and histological categories in the TCGA-THCA cohort.

### 2.14. Statistical Analysis

Group comparisons were conducted using the Kruskal–Wallis test, with pairwise Wilcoxon rank-sum post hoc tests. Pearson correlation coefficients were computed to assess associations among continuous variables. As a quality-control measure, we independently recalculated the TDS for all TCGA-THCA samples and confirmed high concordance with the official TCGA-THCA TDS values using Spearman’s rank correlation. All analyses were performed in R (version 4.2.2) using the tidyverse, ggpubr, and rstatix, pROC packages. Statistical significance was set at *p* < 0.05.

## 3. Results

### 3.1. Clinicopathological Characteristics of the Discovery Cohort

The discovery cohort included 10 papillary TC samples and 9 NG samples. In the thyroid cancer group, the median age was 39 years (range: 16–61), with females comprising 70%. Most cases were classical papillary TC (*n* = 8, 80%), along with two cases of follicular variant. No lymph node metastases were identified in any thyroid carcinoma sample. Tumour size ranged from 0.4 to 5.0 cm (median 1.5 cm). In the goitre group, the median age was 44 years (range 37–62), all patients being female. All cases were diagnosed with euthyroid nodular goitre (*n* = 8), with nodule sizes ranging from 2.0 to 7.0 cm (median 3.0 cm). Age and sex distributions did not differ between the papillary TC and NG groups. The clinicopathological characteristics of the patients were presented in Table 1 and Table 2.

### 3.2. Tumour Microenvironment Classification and Immune Landscape of Thyroid Carcinoma

**Figure 1:**
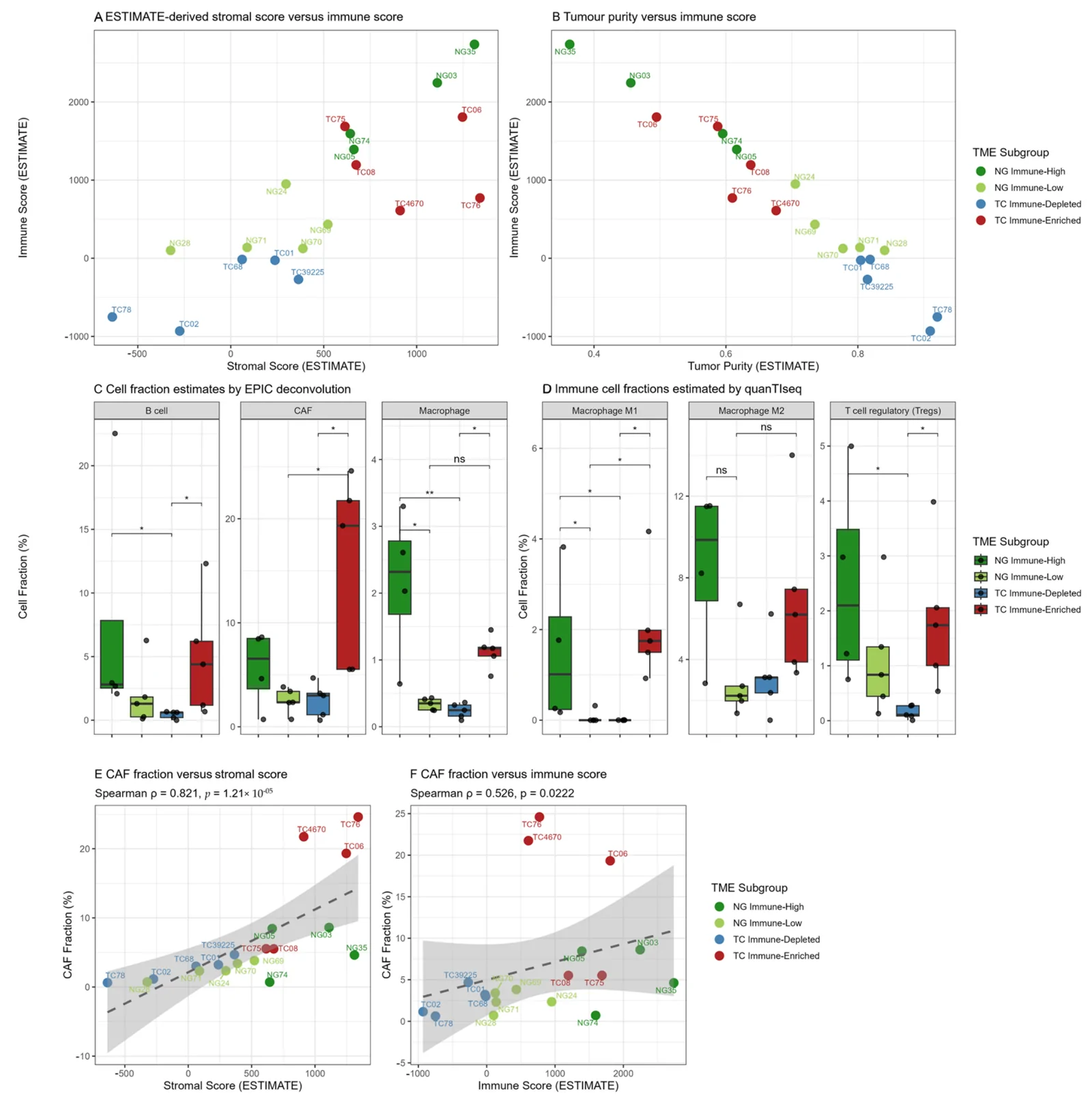
TME Classification and Immune Landscape of Thyroid Carcinoma. (**A**) ESTIMATE-derived stromal score versus immune score for all 19 samples. Samples cluster into four distinct TME subgroups defined by independent median stratification within malignant (TC) and non-malignant (NG) cohorts: TC Immune-Enriched (TC-IE, red, *n* = 5), TC Immune-Depleted (TC-ID, blue, *n* = 5), NG Immune-High (NG-IH, dark green, *n* = 4), and NG Immune-Low (NG-IL, light green, *n* = 5). A positive correlation between stromal and immune scores is evident across subgroups. (**B**) Tumour purity versus immune score. A strong negative correlation is observed across all samples (Spearman ρ = −0.967, *p* < 0.0001), with TC-ID samples clustering at the highest purity values (>0.8), consistent with an immune desert phenotype. (**C**) Cell fraction estimates by EPIC deconvolution for significantly differentially infiltrated populations across TME subgroups (Kruskal–Wallis, *p* < 0.05): B cells, Cancer-Associated Fibroblasts (CAFs), and Macrophages. TC-IE tumours are characterised by markedly elevated CAF infiltration (mean 15.3% ± 9.2%) compared with TC-ID (2.5% ± 1.7%) and NG-IL (2.5% ± 1.2%). Pairwise comparisons by Dunn post hoc test with Benjamini–Hochberg correction; * *p* < 0.05, ** *p* < 0.01, ns = not significant. (**D**) Immune cell fractions estimated by quanTIseq for significantly differentially infiltrated populations: Macrophage M1, Macrophage M2, and T regulatory cells (Tregs). (**E**) Scatter plot of CAF fraction versus ESTIMATE-derived stromal score across all samples. A strong positive correlation is observed (Spearman ρ = 0.821, *p* < 0.0001). (**F**) Scatter plot of CAF fraction versus ESTIMATE-derived immune score. A moderate positive correlation (Spearman ρ = 0.526, *p* = 0.022) reflects co-enrichment of CAFs and immune infiltrates in TC-IE tumours.

Within the TC and NG groups separately, samples were split at the median ESTIMATE immune score, giving four TME subgroups. Thresholds were established separately for the malignant and non-malignant groups (Figure 1A). In the carcinoma group, the median immune score was 297.9, with groups TC-IE (*n* = 5; scores ranging from 610 to 1807) and TC-ID (*n* = 5; scores from −932 to −25). In the goitre group, the median score was 950.4, with NG-IH (*n* = 4; scores ranging from 1393 to 2738) and NG-IL (*n* = 5; scores ranging from 101 to 950). Significant differences among groups were identified (Kruskal–Wallis χ^2^ = 15.3, *p* = 0.0016). Dunn’s test showed that TC-ID was significantly different from TC-IE (*p* = 0.010) and NG-IH (*p* = 0.002). However, TC-IE and NG-IH did not differ significantly (*p* = 0.412), indicating similar levels of immune infiltration.

A strong negative correlation was observed between the immune score and tumour purity across all samples (Spearman ρ = −0.967, *p* < 0.0001; Figure 1B), indicating that increased immune infiltration is associated with lower tumour cell content. TC-ID tumours were grouped at the highest tumour purity level, with significant differences among subgroups (Kruskal–Wallis χ^2^ = 15.0, *p* = 0.0018). Purity is reported as a descriptive covariate; for NG, it denotes the non-immune, non-stromal fraction. Moreover, TC-ID samples had significantly higher purity than TC-IE (*p* = 0.010) and NG-IH (*p* = 0.005).

### 3.3. Cellular Composition of the TME

Immune cell fraction analysis using quanTIseq identified significant differences across TME subgroups for Macrophage M1 (Kruskal–Wallis χ^2^ = 13.60, *p* = 0.0035), Macrophage M2 (χ^2^ = 8.40, *p* < 0.038), and T regulatory cells (Tregs; χ^2^ = 10.52, *p* = 0.015; Figure 1D). TC-ID tumours showed an almost complete absence of M1 macrophages, whereas TC-IE and NG-IH exhibited comparable M1 macrophage levels. Treg fractions followed the pattern NG-IH > TC-IE >> TC-ID, suggesting a regulatory T-cell component in immune-enriched tumours (Figure 1D) [38].

EPIC-based cellular abundance profiling also revealed variability in immune and stromal components among TME subgroups. Significant differences were noted in B cells (Kruskal–Wallis χ^2^ = 9.35, *p* = 0.025), CAFs (χ^2^ = 10.1, *p* = 0.018), and Macrophages (χ^2^ = 14.4, *p* = 0.002; see Figure 1C). Pairwise tests showed that TC-IE was markedly different from both NG-IL (Dunn test, *p*adj = 0.021) and TC-ID (*p*adj = 0.021), whereas no notable differences were observed among the other three subgroups.

NG-IH samples were characterised by broad immune cell enrichment, including high CD8+ T cell (mean: 11.4% ± 2.1%) and B cell fractions, consistent with chronic lymphocytic infiltration. In contrast, TC-IE samples showed a distinct stromal signature with markedly elevated CAF infiltration (mean: 15.3% ± 9.2%) compared with TC-ID (2.5% ± 1.7%), NG-IL (2.5% ± 1.2%), and NG-IH (5.6% ± 3.7%) (Figure 1C,D). This pattern suggests that fibroblast-driven stromal remodelling—rather than classical lymphocytic infiltration—may represent a defining feature of immune-enriched thyroid tumours.

The CAF cell fraction strongly correlated with the ESTIMATE stromal score (Spearman ρ = 0.821, *p* < 0.0001), consistent with CAFs being a major cellular contributor to increased stromal signals (Figure 1E). CAF levels were moderately linked to the immune score (ρ = 0.526, *p* = 0.022) and negatively associated with tumour purity (ρ = −0.642, *p* = 0.004), suggesting that CAF-rich TC-IE tumours generally have lower purity and higher immune infiltration (Figure 1F). MCP-counter fibroblast scores closely matched EPIC-derived CAF estimates (ρ = 0.870, *p* < 0.0001), supporting the reliability of the fibroblast signal across different deconvolution methods.

### 3.4. Differential Expression Analysis

**Figure 2:**
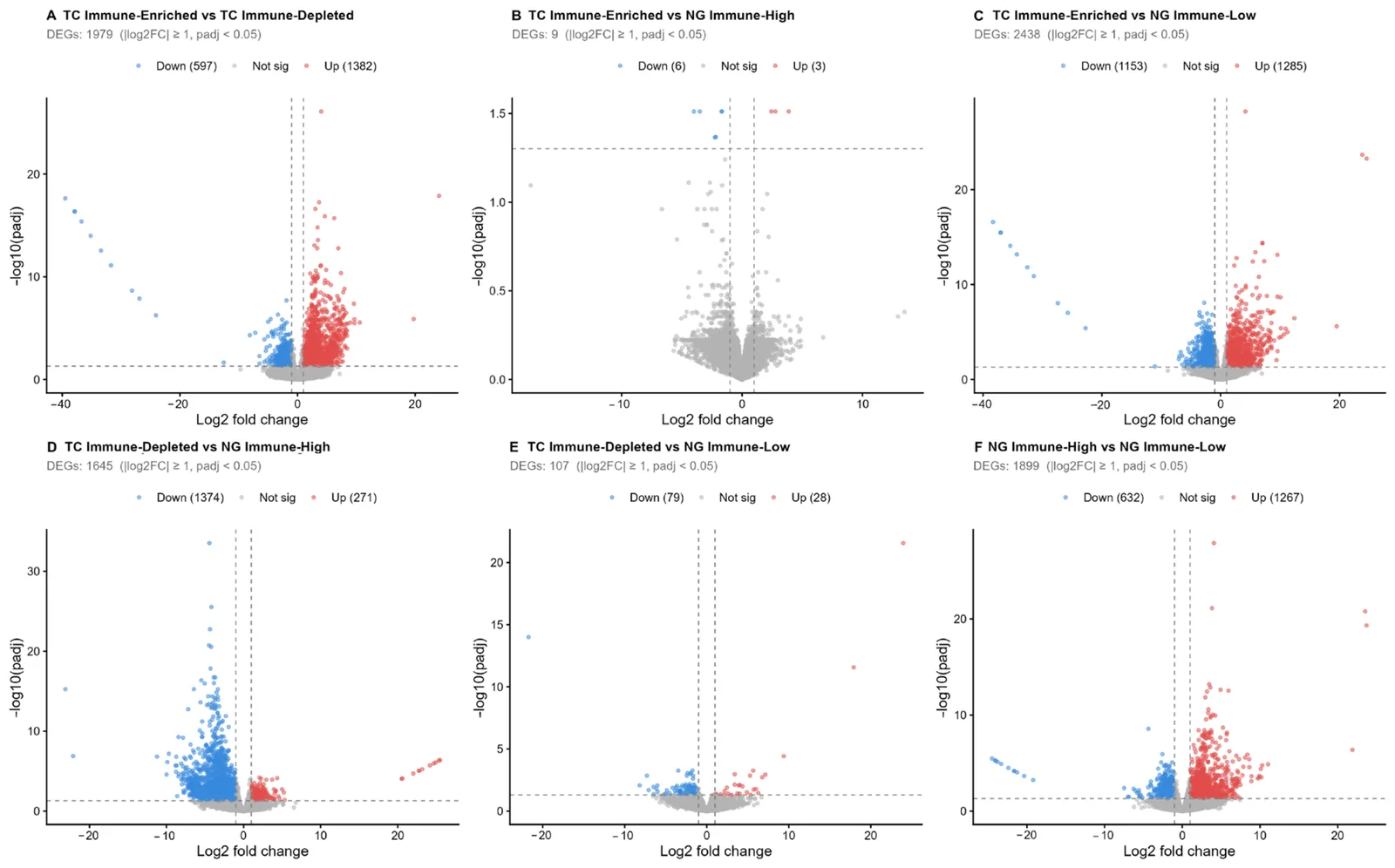
Differential gene expression across six pairwise TME subgroup comparisons. Volcano plots displaying differentially expressed genes (DEGs) for each of the six pairwise comparisons between TME subgroups (**A**–**F**). The horizontal dashed line indicates the adjusted *p*-value threshold (*p*adj < 0.05); the vertical dashed lines indicate |log2FC| ≥ 1. Red dots: significantly upregulated genes; blue dots: significantly downregulated genes; grey dots: non-significant. Total DEG counts and directional breakdown are shown above each plot.

Differentially expressed genes (DEG) analysis indicated that transcriptional identity was driven primarily by the immune phenotype rather than histopathology (Figure 2A–F). Unsupervised hierarchical clustering of 455 genes identified across all four pairwise contrasts recapitulated the TME classification without prior group information, with immune activation status, rather than malignancy, emerging as the primary axis of transcriptomic variation. The transcriptomic convergence between TC Immune-Enriched and NG Immune-High was remarkable, with only nine DEGs distinguishing these two immune-activated states (three upregulated—*HPCAL4*, *GABBR2*, and *MYRF*; six downregulated—*NPL*, *DES*, *LTF*, *SNHG15*, *FABP5*, and *FTL*; Figure 2B).

**Figure 3:**
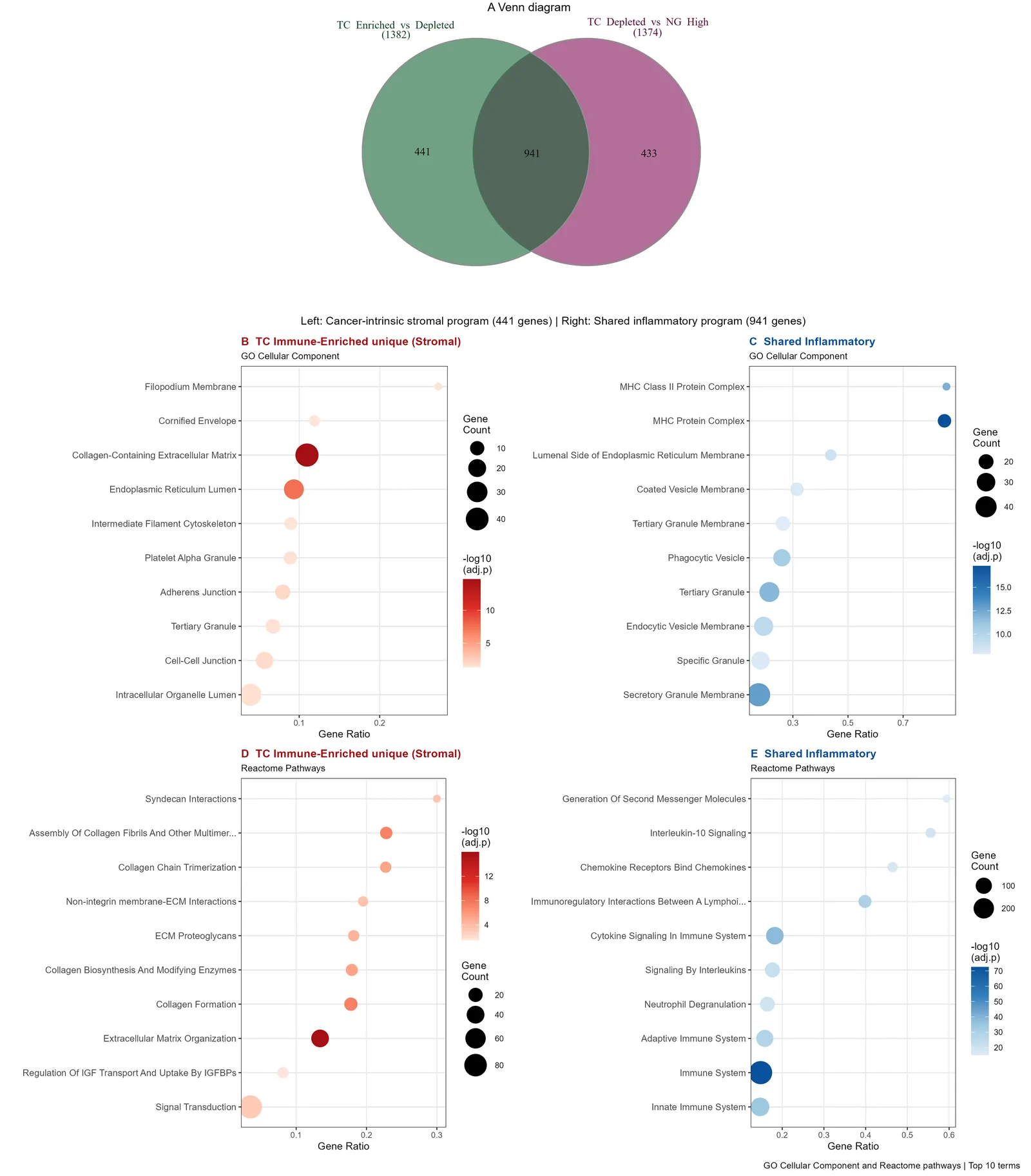
Intersection analysis identifies tumour-associated stromal and shared inflammatory transcriptional programmes. (**A**) Venn diagram showing the overlap between upregulated DEGs from TC Immune-Enriched vs. TC Immune-Depleted (1382 genes) and TC Immune-Depleted vs. NG Immune-High (1374 genes). The 441 unique genes define a tumour-associated stromal programme; the 941 shared genes define a shared inflammatory programme. (**B**,**C**) GO Cellular Component enrichment dot plots for the tumour-associated stromal programme (**B**) and shared inflammatory programme (**C**), showing top 10 terms. Dot size represents gene count; colour intensity represents -log10(adjusted *p*-value). (**D**,**E**) Reactome pathway enrichment dot plots for the tumour-associated stromal programme (**D**) and shared inflammatory programme (**E**), showing top 10 terms. The stromal programme is characterised by collagen-containing extracellular matrix organisation and ECM remodelling pathways; the inflammatory programme is characterised by innate and adaptive immune signalling.

To separate tumour-associated stromal programme from inflammation-driven signals, we intersected 1382 genes upregulated in TC-IE versus TC-ID with 1374 genes upregulated in NG-IH versus TC-ID. The 941-gene intersection indicated a shared inflammatory programme in malignant and benign immune-activated tissue. The remaining 441 genes, which met the DEG criteria only in the TC-IE comparison, constitute a candidate tumour-associated stromal programme (Figure 3A; Supplementary Table S1).

Functional enrichment showed complete orthogonal separation between the two gene sets. The 441 TC-unique genes were enriched for Collagen-Containing Extracellular Matrix and Extracellular Matrix Organisation (Figure 3B,D), while the 941 shared genes were enriched for MHC Protein Complex and Immune System, with no overlap between categories (Figure 3C,E).

Within the Collagen-Containing ECM GO term, 22 genes exceeded the threshold and showed increased expression in TC-IE. This set comprises collagens (*COL1A1*, *COL1A2*, *COL3A1*, *COL5A1*, *COL8A1*, *COL10A1*, *COL11A1*, *COL13A1*), matrix proteins (*THBS1*, *THBS2*, *POSTN*, *VCAN*), cross-linking enzymes (*LOXL1*, *LOXL2*), and CAF markers (*CTHRC1*, *LUM*, *ASPN*, *EMILIN2*, *FIBCD1*). These findings are consistent with TGFβ-associated stromal remodelling [39]. These genes met the DEG criteria only in the carcinoma comparison, but this does not show that the process is absent from immune-activated benign tissue.

### 3.5. Protein-Protein Interaction Results

To further examine functional connectivity within the 441-gene tumour-associated stromal programme, we performed STRING protein-protein interaction analysis. Of the 441 input genes, 416 were mapped to human protein nodes. At a high-confidence interaction threshold (combined score ≥ 0.700), the network contained 239 edges, compared with 78 expected edges, indicating significant protein-protein interaction enrichment (PPI enrichment *p* < 1.0 × 10^−16^; Supplementary Figure S1). The network was dominated by an ECM-related cluster of fibrillar collagens, matricellular proteins, and matrix-remodelling enzymes, including ADAMTS2, ASPN, COL11A1, COL1A1, COL1A2, COL3A1, COL5A1, COL5A2, COL6A3, CTHRC1, IBSP, LUM, POSTN, SPARC, THBS2, and ZNF469.

Functional enrichment of the STRING network was consistent with an ECM-centred network. The most significantly enriched biological processes included extracellular matrix organisation (33 genes; FDR = 3.0 × 10^−11^), extracellular encapsulating structure organisation (34 genes; FDR = 1.89 × 10^−11^), and collagen fibril organisation (13 genes; FDR = 5.22 × 10^−6^). Cellular-component analysis showed strong enrichment for extracellular matrix (51 genes; FDR = 5.10 × 10^−17^), collagen trimer (16 genes; FDR = 7.94 × 10^−8^), and fibrillar collagen trimer (7 genes; FDR = 8.51 × 10^−6^). Reactome analysis further identified extracellular matrix organisation (41 genes; FDR = 1.75 × 10^−15^), ECM proteoglycans (16 genes; FDR = 7.82 × 10^−8^), and collagen formation (16 genes; FDR = 3.51 × 10^−7^). The network was also enriched for the Hallmark epithelial-mesenchymal transition gene set (35 genes; FDR = 1.77 × 10^−18^). These results indicate that the genes in the programme are more densely interconnected than expected by chance and are organised around collagen-rich ECM, consistent with a functionally coherent module.

### 3.6. WGCNA Results

**Figure 4:**
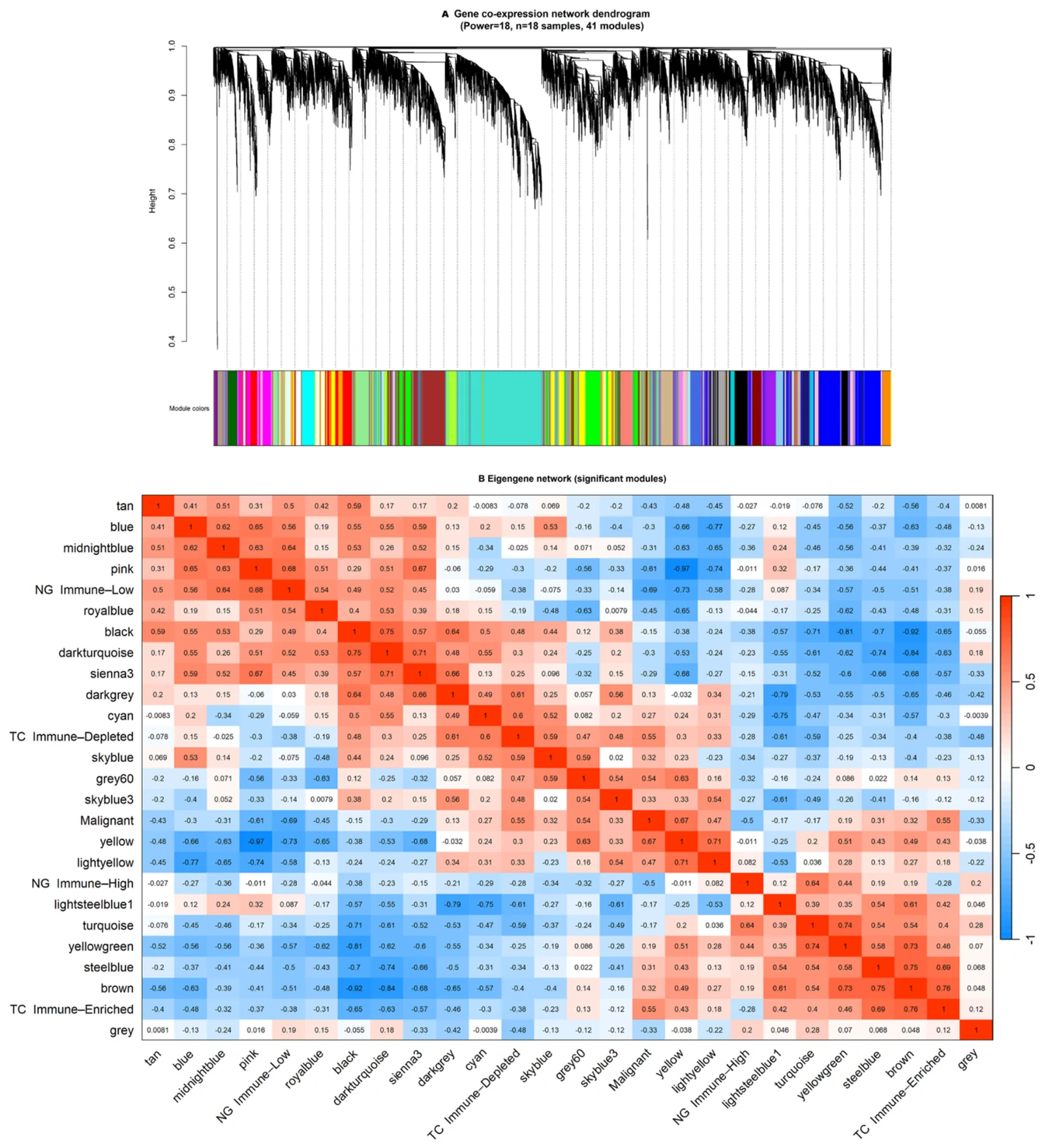
WGCNA gene co-expression network structure. (**A**) Dendrogram of 17,868 genes based on topological overlap dissimilarity. Branches show co-expression modules; the coloured bar below indicates 41 modules identified by dynamic tree cutting, with colours representing different modules (grey for 88 unassigned genes). (**B**) Heatmap of module eigengene correlations with four tumour microenvironment (TME) traits and malignancy status, focusing on 21 modules significantly associated with at least one trait (*p* < 0.05). Cell values are correlation coefficients; colour from −1 (blue) to +1 (red). Modules and traits are ordered by hierarchical clustering. Steelblue and brown modules relate to the TC Immune-Enriched trait; black and dark turquoise relate in the opposite direction.

WGCNA of 17,868 genes across 18 samples (after excluding one outlier) identified 41 co-expression modules, with only 88 genes remaining unassigned (Figure 4A). Module-trait correlation analysis revealed 21 modules significantly associated with the four TME subgroups and malignancy (*p* < 0.05). The modules most positively correlated with the TC-IE phenotype were the brown module (1148 genes; r = 0.757, *p* = 3 × 10^−4^) and the steelblue module (154 genes; r = 0.689, *p* = 0.0016). In contrast, the black (743 genes; r = −0.648, *p* = 0.0036) and dark turquoise (255 genes; r = −0.631, *p* = 0.005) modules were negatively correlated with TC-IE, indicating that these gene programmes are comparatively suppressed in immune-enriched tumours (Figure 4B). In addition to the stromal steelblue module, the brown module showed a strong positive correlation with the TC-IE phenotype. Its hub genes, *NECTIN4*, *TACSTD2*, *LIPH* and *DPP4*, were consistent with an epithelial tumour transcriptional programme. Given this study’s focus on TME stromal remodelling, we did not investigate the brown module further.

Functional enrichment of the steelblue module revealed exclusive enrichment for Extracellular Matrix Organisation (GO:0030198; *p*adj = 6.1 × 10^−20^), Collagen Fibril Organisation (GO:0030199; *p*adj = 2.2 × 10^−13^), and Collagen-Containing Extracellular Matrix (GO:0062023; *p*adj = 7.0 × 10^−27^), consistent with an ECM remodelling programme. In contrast, the black module-negatively correlated with TC Immune-Enriched—was enriched for Thyroid Hormone Metabolic Process (GO:0042403; *p*adj = 0.011) and Axoneme Assembly (GO:0035082; *p*adj = 3.2 × 10^−4^), consistent with loss of thyroid differentiation and ciliary function in immune-activated malignant tissue.

Hub gene analysis of the steelblue module identified *CTHRC1* (MM = 0.973), *INHBA* (MM = 0.962), *COL3A1* (MM = 0.956), *LAMP5* (MM = 0.951), and *LINC01614* (MM = 0.945) as the most highly connected genes. Comparison with the DESeq2 intersection analysis showed that all 20 steelblue hub genes were in the 441-gene set (100% overlap), and 51 of 154 steelblue module genes (33.1%) were also in the set. Because both analyses use the same samples, this indicates internal consistency rather than independent replication and supports the steelblue module as a major component of the tumour-associated stromal programme in TC Immune-Enriched tumours.

The cyan module (*n* = 373 genes; r = 0.604, *p* = 0.0079) was linked to the TC-ID phenotype and showed no significant association with any other TME subgroup (Figure 4). Functional enrichment analysis revealed that the main pathways involved mRNA splicing via the spliceosome, E2F-driven DNA replication, and the assembly of the ORC complex at the origin of replication.

The dark grey module (*n* = 243 genes; r = 0.605, *p* = 0.0078) was also positively associated with the TC-ID trait (Figure 4). Enrichment analysis identified pathways such as fatty acid metabolism, branched-chain amino acid catabolism, sphingolipid biosynthesis, and ATF6-mediated chaperone activation. Hub genes such as *SLC25A15*, *ETFDH*, and *COQ9*, linked to mitochondrial transport, along with *VAPB*, involved in ER–mitochondrial contact sites, were notable. Both modules indicate that TC-ID tumours exhibit a consistent proliferative programme and lipid- and amino acid-based metabolic self-sufficiency. In other tumour types, similar configurations have been associated with decreased immunogenicity and metabolically supported immune evasion, rather than a robust antitumour immune response [40].

### 3.7. External Evaluation of the Stromal Signature in TCGA-THCA

To assess the 441-gene stromal signature in an independent cohort, we applied ssGSEA-based scoring to 456 papillary thyroid carcinoma samples from the TCGA-THCA dataset. Before applying stromal scoring, we ensured methodological consistency by recalculating the TDS and comparing it with the values reported in the official TCGA paper. This comparison resulted in a Pearson correlation of r = 0.972 (*p* < 2.2 × 10^−16^, *n* = 361), confirming the reliability of our computational approach.

### 3.8. Molecular Associations of Stromal ssGSEA Score

**Figure 5:**
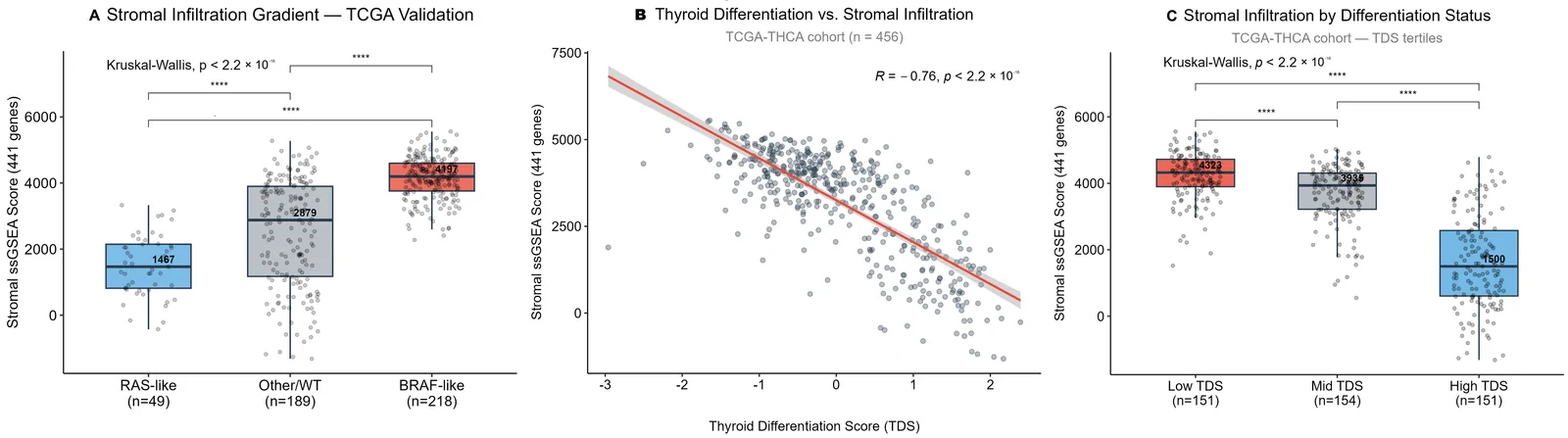
TCGA-THCA evaluation: molecular associations of the stromal signature. (**A**) Stromal ssGSEA scores stratified by BRAF/RAS molecular subtype in the TCGA-THCA cohort (*n* = 456). BRAF-like tumours show significantly higher stromal enrichment than RAS-like and Other/WT tumours (Kruskal–Wallis *p* < 2.2 × 10^−16^). The median score is shown within each boxplot. Significance brackets indicate pairwise comparisons (**** *p* < 0.0001). (**B**) Scatter plot showing the correlation between Thyroid Differentiation Score (TDS) and stromal ssGSEA score across all 456 samples. Pearson r = −0.76, *p* < 2.2 × 10^−16^. The red line indicates the linear regression fit with a 95% confidence interval. (**C**) Stromal ssGSEA scores stratified by TDS tertile (Low, Mid, High; *n* = 151 per tertile). The lowest-differentiation tertile shows significantly higher stromal enrichment than the mid and high tertiles (Kruskal–Wallis *p* < 2.2 × 10^−16^).

The stromal ssGSEA score indicated a strong negative correlation with the TDS across the cohort (Pearson r = −0.76, *p* < 2.2 × 10^−16^, *n* = 456), indicating that higher stromal-programme enrichment is associated with lower thyroid differentiation (Figure 5B). Stratifying samples into TDS tertiles revealed a clear gradient: the lowest-differentiation tertile had significantly higher stromal scores (median 4323) than the middle (3935) and highest tertiles (1500; Kruskal–Wallis *p* < 2.2 × 10^−16^; Figure 5C). Stromal scores also differed significantly across *BRAF* V600E/*RAS* molecular subtypes, with *BRAF*-like tumours showing the highest stromal enrichment (median 4197), followed by Other/WT (2879) and RAS-like tumours (1467; Kruskal–Wallis *p* < 2.2 × 10^−16^; Figure 5A).

### 3.9. Clinical Associations of Stromal ssGSEA Score

**Figure 6:**
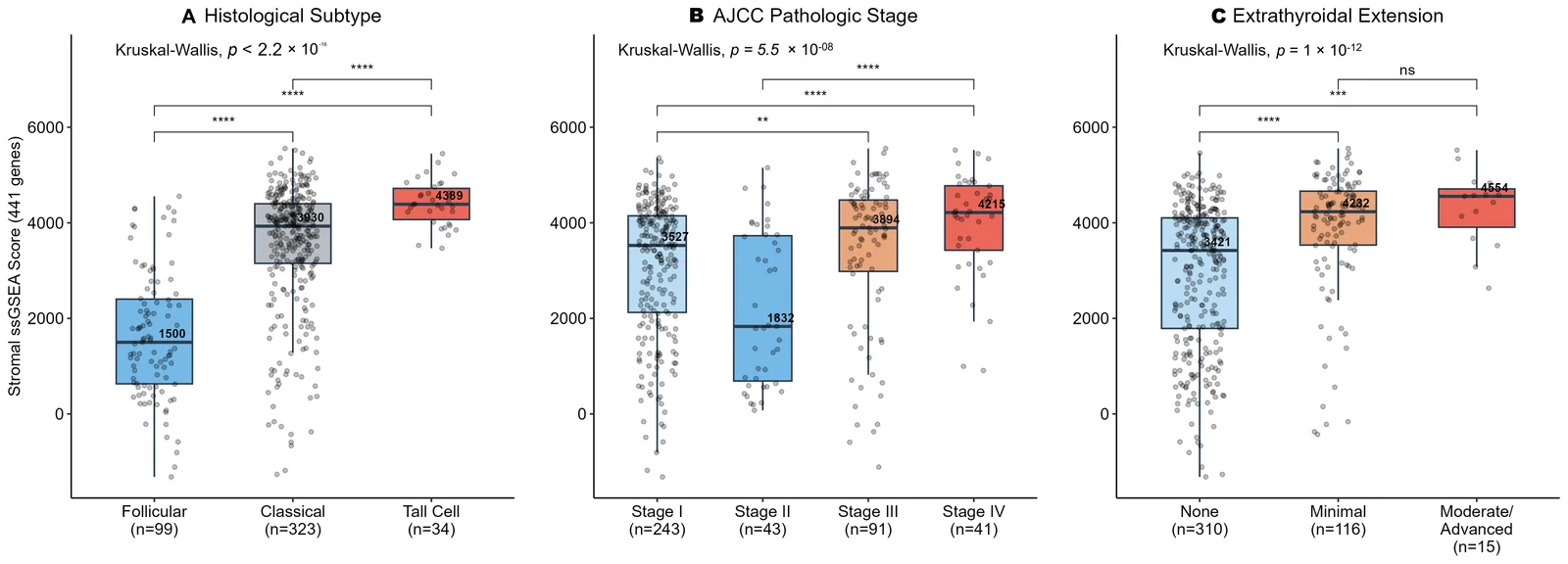
TCGA-THCA evaluation: clinical associations of the stromal signature. (**A**) Stromal ssGSEA scores stratified by histological subtype. The tall cell variant shows the highest stromal enrichment (median 4389), followed by classical (3990) and follicular (1500) subtypes (Kruskal–Wallis *p* < 2.2 × 10^−16^). (**B**) The stromal scores differ significantly across AJCC stages (Kruskal–Wallis *p* = 5.5 × 10^−8^); Stage II tumours show lower enrichment than Stage I, consistent with age-based upstaging criteria in differentiated thyroid cancer. (**C**) Extrathyroidal extension status in the TCGA-THCA cohort: the stromal enrichment increases progressively with extrathyroidal extension severity, from no extension (median 3421) through minimal (4232) to moderate/advanced extension (4554; Kruskal–Wallis *p* = 1 × 10^−12^). Median scores are shown within each boxplot. Significance brackets indicate pairwise Dunn test results; ns = not significant, ** *p* < 0.01, *** *p* < 0.001, **** *p* < 0.0001.

Stromal enrichment differed significantly across histological subtypes (Kruskal–Wallis χ^2^ = 133.92, *p* < 2.2 × 10^−16^), with tall cell variant exhibiting the highest scores (median 4389), followed by classical (3990) and follicular (1500) subtypes, consistent with the established aggressiveness hierarchy of papillary TC variants (Figure 6A). AJCC pathologic stage was significantly associated with stromal enrichment (Kruskal–Wallis χ^2^ = 36.65, *p* = 5.45 × 10^−8^). Stage I and III-IV tumours showed higher stromal scores (medians 3527 and 3894–4215, respectively) compared with Stage II (1832) (Figure 6B). The lower stromal enrichment in Stage II likely reflects the age-based staging criterion in differentiated thyroid cancer, whereby patients older than 55 years are automatically upstaged regardless of tumour biology [41].

Another clinical feature, such as extrathyroidal extension, was also associated with progressively higher stromal ssGSEA score (Kruskal–Wallis χ^2^ = 55.17, *p* = 1.05 × 10^−12^; Figure 6C): tumours without extension had a median score of 3421, rising to 4232 for minimal extension and 4554 for moderate/advanced extension, suggesting that stromal remodelling may contribute to invasive tumour behaviour.

### 3.10. ROC Analysis

**Figure 7:**
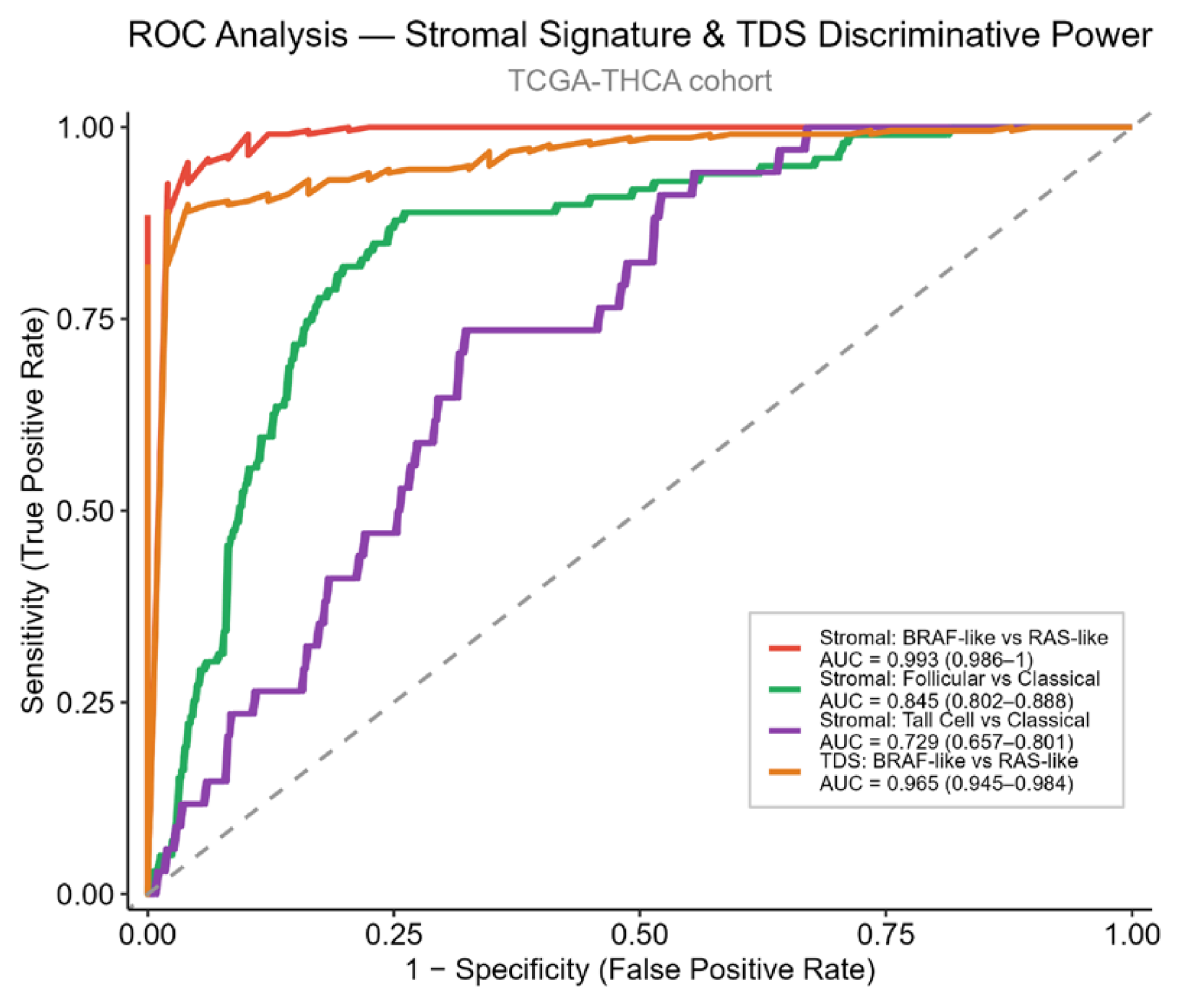
ROC analysis of stromal signature and TDS discriminative power in TCGA-THCA. The stromal enrichment score was strongly associated with BRAF-like and RAS-like molecular subtypes (AUC = 0.993, 95% CI: 0.986–1.000; red), outperforming the TDS in the same comparison (AUC = 0.965, 95% CI: 0.945–0.984; orange). The signature also effectively distinguished follicular from classical histological subtypes (AUC = 0.845, 95% CI: 0.802–0.888; green) and showed moderate discrimination between tall cell and classical subtypes (AUC = 0.729, 95% CI: 0.657–0.801; purple). The diagonal dashed line represents chance-level discrimination.

ROC analysis was used to assess how cleanly the stromal signature separates established molecular and histological categories, serving as a measure of biological signal strength rather than a proposed diagnostic application (Figure 7). The stromal ssGSEA score showed high discriminative performance between *BRAF*-like and *RAS*-like molecular subtypes (AUC = 0.993, 95% CI: 0.986–1.000), outperforming even the TDS in the same comparison (AUC = 0.965, 95% CI: 0.945–0.984; Figure 7). This finding indicates that stromal enrichment is strongly associated with the BRAF-like expression subtype. The ROC analysis is presented as a descriptive measure of molecular separation within this reference cohort and not as validation of a clinical diagnostic test.

The signature also effectively distinguished follicular from classical histological subtypes (AUC = 0.845, 95% CI: 0.802–0.888; Figure 7), with follicular tumours exhibiting markedly lower stromal enrichment despite their well-differentiated phenotype. The discrimination between tall cell and classical subtypes was moderate (AUC = 0.729, 95% CI: 0.657–0.801; Figure 7), likely reflecting the limited sample size of the tall cell group (*n* = 34) within the TCGA cohort. Collectively, these findings support the stromal signature as a marker that captures biologically meaningful variation across molecular subtypes and histological categories in an independent cohort of 456 papillary TC samples.

## 4. Discussion

The limited differential expression between TC Immune-Enriched tumours and NG Immune-High tissue—with only nine genes meeting the differential-expression criteria-suggests substantial transcriptomic similarity between these malignant and benign thyroid groups. This has a direct implication for TME-based biomarker strategies: immune infiltration scores in isolation may reflect the intensity of immune activation rather than its malignant context [42].

The nine genes meeting the differential-expression criteria provide preliminary clues to the differences between these transcriptionally similar groups. The three genes upregulated in TC-IE—*HPCAL4*, *GABBR2*, and *MYRF*—have established roles in neural and glial biology, raising the possibility of altered neuro-immune or neuro-stromal interactions in malignant immune-enriched tissues [43,44,45]. Conversely, reduced expression of *DES*, *LTF*, and *FTL* points to differences in cytoskeletal or stromal features, innate immune activity, and iron handling between the malignant and benign groups [46,47,48]. These observations remain hypothesis-generating and require independent validation.

Limited differential expression between TC-IE and NG-IH suggests a shared inflammatory signal, although small subgroup sizes limit interpretation. The 941 overlapping genes were enriched for immune-related terms (Figure 3C,E), whereas the 441 genes significant only in the carcinoma comparison were enriched for extracellular-matrix organisation, defining a candidate tumour-associated stromal programme. However, significance in only one comparison does not establish a TC-IE-NG-IH difference or malignant-benign discrimination.

TGFβ- and ECM-remodelling pathways were enriched in TC-IE tumours, consistent with a tumour-associated TGFβ axis, which drives CAF activation, collagen deposition, and matrix stiffening—processes widely implicated in malignant stromal remodelling rather than inflammatory infiltration alone [49,50,51]. In TCGA-THCA, stromal enrichment scores were strongly correlated with loss of thyroid differentiation and a BRAF-like molecular identity. This suggests that ECM remodelling is associated with the BRAF-like transcriptional state of papillary TC, although a link to oncogenic TGFβ signalling is plausible but was not tested [52,53,54].

Notably, despite their designation as immune-enriched, TC Immune-Enriched tumours were characterised by markedly elevated CAF infiltration rather than lymphocytic dominance. The CAF-over-lymphocyte pattern raises the possibility that extensive stromal remodelling contributes to an immune-excluded microenvironment. However, because bulk RNA sequencing and computational deconvolution cannot establish spatial relationships between CAFs and lymphocytes, this interpretation remains hypothetical and requires spatial or histological validation [55,56]. This distinction is clinically relevant, as CAF-rich immune-enriched tumours may respond differently to immunotherapy than tumours with genuine lymphocytic infiltration [57]. Evaluation in TCGA-THCA, discussed below, adds associative context but does not test this mechanism.

Within the tumour-associated stromal module, *CTHRC1*, *INHBA*, and *COL3A1* emerged as hub genes, further anchoring the TC-IE programme to established CAF biology. *CTHRC1* encodes a secreted remodelling factor recently identified as a defining marker of CAF subsets across multiple solid tumours, where it promotes cancer cell invasion and EMT via Wnt/β-catenin, PI3K/AKT, and LIF-STAT3 signalling [58,59]. *INHBA*, encoding the activin A subunit, is a canonical CAF-enriched gene whose product drives fibroblast activation and pro-invasive matrix production downstream of stress signalling [60,61]. *COL3A1* is a prototypical fibrillar collagen co-expressed with other matricellular genes in CAF modules associated with tissue stiffening and poor prognosis across cancer types [62]. Collectively, these hub genes support a *CTHRC1^+^/INHBA^+^/COL3A1^+^* CAF-like stromal programme, a hypothesis that warrants validation by spatial transcriptomics or CAF-specific profiling.

The immune-depleted cluster was characterised by coordinated expression of mitochondrial and ER-mitochondrial genes, including *SLC25A15*, *ETFDH*, *COQ9* and *VAPB*, suggesting a distinct tumour-intrinsic metabolic state. This profile is consistent with an immune-desert phenotype, in which limited immune infiltration reflects low tumoural immunogenicity rather than active exclusion [63,64].

Evaluation in 456 TCGA-THCA samples showed that the 441-gene programme is associated with molecular and clinical variation across papillary TC. The strong inverse correlation between stromal enrichment and the TDS (r = −0.76, *p* < 2.2 × 10^−16^) positions stromal remodelling as a transcriptional counterpart to dedifferentiation, and is consistent with a model in which BRAF-MEK-ERK signalling contributes to both loss of thyroid differentiation and activation of TGFβ-associated stromal programmes [15]. In thyroid cancer, BRAF V600E has been shown to induce secretion of functional TGFβ and repress thyroid differentiation genes through an autocrine TGFβ/Smad loop, while in colorectal cancer and melanoma analogous BRAF-TGFβ-CAF circuits have been implicated in CAF activation and ECM remodelling, supporting the plausibility of this pathway in papillary TC [15,65,66]. In the TCGA-THCA dataset, this model is supported by markedly higher stromal scores in BRAF-like tumours (median 4197) than in RAS-like tumours (median 1467). Concordantly, the consistently low stromal enrichment of follicular variant papillary TC across the TCGA-THCA cohort independently recapitulates the known RAS-like molecular identity of this subtype, suggesting that the stromal signature reflects oncogenic context rather than histological architecture alone.

The association between stromal enrichment and extrathyroidal extension—with scores rising progressively from no extension (median 3421) through minimal (4232) to moderate/advanced extension (4554)—is consistent with a role for ECM remodelling in invasive tumour behaviour, in line with the established function of CAF-derived matrix in facilitating basement membrane disruption and local invasion [55].

The apparently paradoxical finding of lower stromal enrichment in Stage II than Stage I tumours (medians 1832 vs. 3527) warrants specific consideration. Under AJCC 8th edition criteria, patient age ≥ 55 years substantially influences stage assignment in differentiated thyroid cancer, meaning Stage II tumours are not inherently more aggressive; they occur in older patients, who frequently harbour follicular variant or *RAS*-like disease—both characteristically low-stromal in our data. This anomaly illustrates a broader limitation of anatomical staging systems in capturing biological heterogeneity in thyroid cancer, and raises the possibility that molecular or stromal enrichment scores could complement stage as integrative prognostic variables—a hypothesis that survival outcome analyses in adequately powered cohorts could directly address.

These findings are biologically relevant and hypothesis-generating, but they do not establish diagnostic or prognostic utility. The similarity between malignant and benign immune-rich thyroid tissue cautions against using aggregate immune scores alone as markers of malignancy. In TCGA-THCA, the ECM/CAF-related stromal programme was associated with lower thyroid differentiation, *BRAF*-like identity, tall-cell histology, and extrathyroidal extension. These associations warrant investigation as a potential complement to molecular classification, but they do not demonstrate independent prognostic value. Using them for clinical risk stratification would require validation in independent cohorts with standardised follow-up, recurrence endpoints, and multivariable analyses. Whether they can be reliably detected in preoperative material is also unknown.

This study should be interpreted as a pilot discovery analysis. The discovery cohort is small (*n* = 19), and the TME subgroups include as few as four samples each. This limitation reflects the study’s pilot nature. Because the TCGA-THCA analysis assesses associations within carcinoma only, subgroup-level transcriptomic comparisons require validation in larger, independent cohorts. Second, nodular goitre, while the most clinically relevant non-malignant comparison group available in a surgical cohort, is not a normal thyroid—it is a hyperplastic condition in which immune infiltration, particularly in the NG Immune-High subgroup, may include subclinical autoimmune processes in addition to reactive inflammation. This limits how precisely the shared inflammatory programme can be attributed solely to immune activation. Third, bulk RNA sequencing cannot resolve spatial relationships between cell populations—while deconvolution estimates CAF abundance, it cannot confirm whether CAFs physically encircle tumour nests, co-localise with invasion fronts, or actively exclude cytotoxic lymphocytes. Spatial transcriptomics or multiplex immunohistochemistry in an independent cohort would be required to validate the spatial organisation and functional role of the stromal programme. Fourth, the discovery cohort was not designed or powered for clinicopathological or prognostic analysis, and no recurrences have been recorded, precluding prognostic interpretation. Although large, TCGA-THCA has limited long-term follow-up for disease-specific outcomes in this generally indolent cancer. Fifth, TME subgroups are relative, defined by median ESTIMATE immune scores, and WGCNA included only 18 samples, near the recommended minimum. Finally, TCGA-THCA lacks benign tissue, limiting analyses to associations within carcinoma.

## 5. Conclusions

In conclusion, this exploratory study identified a 441-gene candidate tumour-associated stromal programme enriched for extracellular-matrix organisation and supported by co-expression and interaction-network analyses. *CTHRC1*, *INHBA* and *COL3A1* ranked among the top co-expression hubs. Limited differential expression between immune-enriched papillary thyroid carcinoma and immune-high nodular goitre in this small cohort suggests transcriptomic similarity but does not establish equivalence. This finding cautions against interpreting aggregate immune scores alone as malignancy markers. Among 456 TCGA-THCA papillary thyroid carcinomas, stromal programme enrichment was associated with lower thyroid differentiation, *BRAF*-like identity, tall-cell histology and extrathyroidal extension.

These findings warrant further investigation of stromal remodelling in the thyroid tumour microenvironment but do not establish specificity for malignancy, attribution to a defined CAF subset, a causal role for TGFβ signalling or independent prognostic value. Validation in larger independent cohorts, spatial localisation and functional studies are needed to clarify the programme’s cellular origins and biological significance. Its prognostic potential should be assessed using recurrence endpoints and multivariable analyses.

## Figures and Tables

**Table 1:** Distribution by age, sex and histological variant of the 10 cases with thyroid cancer.

Gender	Number of Patients (*n*)	Percentage (%)					
Male	3	30%					
Female	7	70%					
Age	Number	Percentage (%)					
Below 45 years	5	50%					
Above 45 years	5	50%					
Histological subtype of Papillary thyroid cancer	n	%	Multifocal tumours	Stage I	Stage II	Stage III	Stage IV
Classical variant	8	80%	2	7	0	1	0
Follicular variant	2	20%	0	2	0	0	0
Tumour size							
≤2 cm	8	80%					
>2 cm	2	20%					
Extrathyroidal extension							
With	1	10%					
Without	9	90%					
Surgery							
Total thyroidectomy	6	60%					
Lobectomy	4	40%					
Radioiodine therapy	1	10%					
Close relatives with thyroid disease	*n*	Percentage (%)					
With malignant disease	0	0%					
With benign disease	2	20%					
Without any thyroid disease	8	80%					

**Table 2:** Distribution by age, sex, and histological variant of the 9 cases with nodular goitre.

Gender	Number of Patients (*n*)	Percentage (%)
Male	0	0%
Female	9	100%
Age	Number	Percentage (%)
Below 45 years	7	78%
Above 45 years	2	22%
Total	9	100%
Nodule size, median (range)	3 (2–7) cm	
Surgery		
Total thyroidectomy	4	44%
Partial thyroidectomy	5	56%
Lobe involvement		
Bilateral	5	56%
Unilateral	4	44%
Retrosternal extension		
With	5	56%
Without	4	44%
Close relatives with thyroid disease	*n*	Percentage (%)
With malignant disease	0	0%
With benign disease	1	11%
Without any thyroid disease	8	89%

## Data Availability

The datasets presented in this article are not readily available because the data are part of an ongoing study. Requests to access the datasets should be directed to Radka Kaneva. The R scripts used for the analyses are available at https://github.com/vpetkova5/cancers_tc_transcriptomics (accessed on 9 September 2026).

## Supplementary Materials

The following supporting information can be downloaded at: https://www.mdpi.com/article/10.3390/cancers18203406/s1, Supplementary Table S1. 441-gene set list. Supplementary Figure S1. STRING protein–protein interaction network of the 441-gene tumour-associated stromal programme.

## Abbreviations

The following abbreviations are used in this manuscript:

TC	Thyroid Carcinoma
NG	Nodular Goitre
TME	Tumour Microenvironment
TC-IE	Thyroid Carcinoma Immune-Enriched
TC-ID	Thyroid Carcinoma Immune-Depleted
NG-IH	Nodular Goitre Immune-High
NG-IL	Nodular Goitre Immune-Low
CAF	Cancer-Associated Fibroblast
ECM	Extracellular matrix
TDS	Thyroid Differentiation Score
ssGSEA	Single-sample Gene Set Enrichment Analysis
WGCNA	Weighted Gene Co-expression Network Analysis
BH	Benjamini–Hochberg
DEG	Differential gene expression
